# User profiles in digitalized healthcare: active, potential, and rejecting — a cross-sectional study using latent class analysis

**DOI:** 10.1186/s12913-024-11523-w

**Published:** 2024-09-17

**Authors:** Anja Knöchelmann, Karl Healy, Thomas Frese, Eva Kantelhardt, Rafael Mikolajczyk, Gabriele Meyer, Jan Schildmann, Anke Steckelberg, Max Herke

**Affiliations:** 1 Institute of Medical Sociology, Interdisciplinary Center for Health Sciences, Medical Faculty of the Martin Luther University Halle-Wittenberg, Magdeburger Str. 8, Halle (Saale), 06112 Germany; 2 Institute of General Practice & Family Medicine, Interdisciplinary Center for Health Sciences, Medical Faculty of the Martin Luther University Halle-Wittenberg, Halle (Saale), Germany; 3 AG Global Health, Institute of Medical Epidemiology, Biometry and Informatics, Interdisciplinary Center for Health Sciences, Medical Faculty of the Martin Luther University Halle-Wittenberg, Halle (Saale), Germany; 4 Institute of Medical Epidemiology, Biometry and Informatics, Interdisciplinary Center for Health Sciences, Medical Faculty of the Martin Luther University Halle-Wittenberg, Halle (Saale), Germany; 5 Institute of Health and Nursing Science, Interdisciplinary Center for Health Sciences, Medical Faculty of the Martin Luther University Halle-Wittenberg, Halle (Saale), Germany; 6 Institute for History and Ethics of Medicine, Interdisciplinary Center for Health Sciences, Medical Faculty of the Martin Luther University Halle-Wittenberg, Halle (Saale), Germany

**Keywords:** Digital divide, Digitalized healthcare services, Income, Education, Latent class analysis, User types

## Abstract

**Background:**

There is evidence of different use by different groups of people for general health-related applications. Yet, these findings are lacking for digitalized healthcare services. It is also unclear whether typical use patterns can be found and how user types can be characterized.

**Methods:**

The analyses are based on data from 1 821 respondents to the Health Related Beliefs and Health Care Experiences in Germany panel (HeReCa). Digitalized healthcare services, that were used to determine the user types, include for example sick ﻿notes before/after examination and disease related training. User types were determined by latent class analysis. Individual groups were characterized using multinomial logistic regressions, taking into account socioeconomic and demographic factors as well as individual attitudes towards digitalization in the healthcare system.

**Results:**

Three types were identified: rejecting (27.9%), potential (53.8%) and active (18.3%). Active participants were less likely to be employed, less likely to be highly educated and less skeptical of digital technologies. Potential users were the youngest, most highly-educated and most frequently employed group, with less skepticism than those who rejected. Rejecters were the oldest group, more likely to be female and of higher socio-economic status.

**Conclusions:**

Socio-demographic and socio-economic differences were identified among three user types. It can therefore be assumed that not all population groups will benefit from the trend towards digitalization in healthcare. Steps should be taken to enhance access to innovations and ensure that everyone benefits from them.

**Supplementary Information:**

The online version contains supplementary material available at 10.1186/s12913-024-11523-w.

## Background

Healthcare systems across the world have been facing innovations in the last years, which can be attributed primarily to the increasing importance of digital services. These developments were further fueled by the coronavirus pandemic and are still being driven forward by governments, among others. In Germany, the increase in virtual consultations, the high number of prescribed digital health applications (DiGA), as well as the growing interest in video consultations, online health courses, e-prescriptions and the electronic patient record (ePa) shows that the government’s efforts meet the expectations of the population [1–6]. Providers also show interest in the digitalization of healthcare. In a 2020 survey, two-thirds believed that digital technologies would fundamentally improve medical care for people. Around one fifth are offering their patients digital information sheets before examinations or procedures. About the same amount provide online consultation with an additional 30% claiming this to be a useful service. The same study showed a mixed attitude towards e-prescriptions, with around 60% who do not yet use them but would consider it and 18% who categorically rule them out [7, 8].

However, whether these services are being used is likely to be associated with individual factors. Social differences, which can be observed along the lines of both horizontal (e.g., income or education) and vertical inequality (e.g., age or gender), is summarized under the term *digital divide* and includes three levels [9, 10]. The first focuses on the general access to the necessary technology and the internet. The second divide postulates differences in usage patterns and the individual ability to use digital technologies or navigate the Internet in a targeted manner. The third divide claims that people with varying backgrounds differ in their ability to use digital technologies for improving their health. Research has shown that while the first-level divide is closing, especially in high-resource settings, the second and third still persist [9, 10].

For the use of general digital health services such as fitness trackers, personal health records or searching for information online, studies have already confirmed differences in the user structure. People with a higher socio-economic status, usually measured by education or income, search for health-related information online more frequently or are more likely to use personal health records and fitness trackers [10–20]. No clear trend was found with regard to gender. The most likely differences were found in the search for health-related content and the use of health portals, with women being more probable to use them [17]. There was also an urban-rural difference, with people living in rural areas being more likely to search for health-related information on the internet [13]. With regard to age, studies show that especially younger people use the Internet to search for information or personal health records [11–13, 16–18, 20, 21]. At the same time, older people seem to show great interest in learning digital skills [22, 23].

However, these services are mostly provided by the private sectors and are more oriented towards health promotion than disease management. The extent to which the stated socio-economic and socio-demographic differences can also be found for digitalized healthcare services and whether patterns of (non-)users can be identified has not yet been sufficiently investigated. It should be considered whether these services are reaching the people who are most likely to benefit from the measures due to an increased likelihood of illness or a poorer healthcare availability, such as older people, people with a lower socio-economic status or those in more rural areas. These results can help to optimize the development of future innovations in digitalized healthcare services. The aim of this study is therefore to identify and characterize (non-)user types.

Initial research has shown different usage patterns with regard to digitalized healthcare services. In international studies, younger people and those with higher levels of education were more likely to use telemonitoring services [24]. E-consultations, on the other hand, were more frequently used by older people and those with a lower level of education [25]. For Germany, there are mainly surveys by market and opinion research institutes or health insurance companies, according to which higher-income and younger people as well as those with a higher level of education and from urban areas in particular can imagine using video consultations [2, 16]. Younger people were also more open to using an electronic health record [1].

The studies mentioned have so far been limited to a small number of healthcare services, while other services remain unnoticed but could be helpful in improving the existing gaps in healthcare in rural areas, such as examinations by specialists or digitally including relatives at appointments [26, 27]. These can also be helpful in countering the feared further deterioration in healthcare against the backdrop of demographic change and the associated increase in chronic illnesses in older age in conjunction with the decline in medical staff.

In addition, there has so far been a lack of an overarching view of possible user groups in order to be able to plan suitable interventions and offers in a more targeted manner. The theory of the diffusion of innovations (TDI), which identifies distinct profiles that have different levels of openness to innovation, can be used for a systematic approach [27]: innovators, early adopters, early majority, laggards, non-users. These differ in their ability to understand and apply complex technical knowledge as well as their awareness of innovations and the anchoring in corresponding social structures in which these are exchanged [27, 28]. In terms of their characteristics, the profiles differ from one another, but are very similar in themselves. *Innovators* and *early adopters* usually have a higher socio-economic status than groups of people who adopt innovations later. Innovations thus follow a social gradient in their diffusion [27, 29]. It can be assumed that this also applies to innovations in digitalized healthcare.

Yet, the questions of the extent to which these profiles can also be found for digitalized healthcare services and which factors are related to the likelihood of belonging to one of the groups can be decisive in implementing new services in a target group-oriented manner and to benefit from the natural spread of innovations. The extent to which the individual attitudes of people regarding the opportunities and risks of digitalization in the healthcare sector play a role should also be taken into account. The research questions are therefore: i) Can user types be identified in relation to digitalized healthcare services? ii) Do the user types identified differ in terms of their socio-demographic and socio-economic characteristics? iii) Do the user types identified differ in terms of their attitudes towards technological innovations in the medical sector?

## Methods

### Data

The data basis is the Health Related Beliefs and Health Care Experiences in Germany panel (HeReCa), which was established in 2019 to 2020 in the federal states of Baden-Württemberg, Berlin, North Rhine-Westphalia, Saxony-Anhalt and Schleswig-Holstein [30]. The present study was conducted in July 2020 and includes both new participants (Baden-Württemberg, North Rhine-Westphalia) and those already active (Saxony-Anhalt, Berlin, Schleswig-Holstein). New respondents were recruited via random samples from selected residents' registration offices stratified according to population density. Potential participants were informed of the study once by post and received a link to register, whereupon they were forwarded to the current questionnaire. People who had already registered received an invitation by email and a maximum of two reminder emails. Informed consent was obtained from all of the study participants prior to entering the online survey. The study only included participants of age 18 or older. A total of 1 821 people took part in this panel wave. The study was submitted to and approved by the Ethics Committee of the Medical Faculty of Martin Luther University Halle-Wittenberg (processing no. 2019-044). The questionnaire on digitalization in the healthcare sector was developed as part of the survey (see suppl. material—questionnaire), and the information on socio-economic and socio-demographic characteristics was collected using established instruments in the baseline assessment of the HeReCa-Panel.

### Variables

#### Use of digitalized healthcare services

Respondents were asked to indicate the extent to which they had already used, would use or would not use a total of eleven digitalized healthcare services, including for example follow-up examinations after hospitalization or training on a specific illness (Table 2). These items served as the basis for identifying the different user types.

#### Socio-economic and socio-demographic variables

The demographic variables included sex (male vs. female, diverse was excluded due to too few mentions), age in years, education (university degree vs. no university degree), marital status (married vs. single/ widowed/ divorced), native language (German vs. other), current employment (employed vs. not employed) and size of place of residence (up to 20 000, up to 100 000 and more than 100 000 inhabitants). For education, the dichotomization was set higher due to the above-average level of education of the respondents.

#### Attitudinal variables

Four items on trust in or concern about the use of digital technologies in the medical field were used (Table A1). The response options consisted of a five-point Likert scale from 1 "very low" to 5 "very high". Four items asked the participants about their trust regarding the opportunities for healthcare, organization and administration, treatment of serious illnesses and data-driven improvement of the healthcare system, while four others asked about their concerns regarding the risks to data protection, privacy, a generally negative development in the healthcare system and a poor quality of healthcare apps.

The "Skepticism towards digital technologies in healthcare" scale was created to examine the relationship between attitudes towards the opportunities and risks of digitalized healthcare services and the user types. To form the scale, the four items on trust were inverted and afterwards all items were combined. The scale was used ad hoc due to the good Cronbach's alpha of 0.83, and a factor analysis showed a good fit for a single-factor model.

### Statistical analysis

First, a descriptive overview of the use of digitalized healthcare services was provided. The unweighted sample distribution was compared with a weighted sample distribution in order to detect deviations, in particular due to self-selection and the resulting over- or underrepresentation of certain groups. With the help of weighting, the HeReCa sample was matched to a representative sample from the Federal Statistical Office in terms of age, sex and education (Table A2a, b, c).

Subsequently, a latent class analysis (LCA) was calculated on the unweighted data of the eleven items on the use of digitalized healthcare services in order to identify different classes of users. The R package "poLCA" [31] was used. Latent class analysis can be defined as a finite mixture model that identifies discrete, latent classes with the help of manifest variables [32, 33]. Here it is assumed that the observed variables (e.g., use of e-consultations, e-prescriptions or sick notes) are influenced by unobserved classes or latent classes. These classes represent different groups of individuals who show similar patterns in their responses and are subsequently interpreted as different user types. To identify the classes, five models, each with a different number of classes (two to six), were calculated using the same manifest variables on the use of digitalized healthcare services. The selection of the best number of classes was based primarily on the interpretability of the classes and secondarily on a comparison of the different quality criteria (in particular Bayesian Information Criterion and entropy).

In addition, a multinomial regression was carried out with the different user types as the dependent variable. In a first model, socio-economic and socio-demographic characteristics were considered as independent variables; in a second model, the attitude variables and the federal state were also included. In sensitivity analyses, this model was compared with two separate logistic regressions and an alternative operationalization of education was considered, as well as the sum of previous chronic illnesses.

### Sample description

The majority of participants were married (63.2%), employed (71.2%), a large fraction had a university degree (44.1%) and were rather skeptical about digital technologies in healthcare (mean: 3.1). More than half were female (54.0%). With the exception of education, there were only minor differences between the unweighted and weighted sample (Table 1). An average of 5% item non-response was observed, excluding items without any missing values due to the survey mode (federal state, place of residence and native language). An analysis of the patterns of missing values revealed a unit non-response of 2.5% and an overall low probability of a significant bias due to missing values.

**Table 1:** Sample description

**Feature**	**Unweighted**	**Weighted**^c^
Age		
Mean value (standard deviation)^a^	51.8 (15.1)	50.4 (16.7)
Missing	4.8%	0.0%
Sex		
Male	43.7%	49.8%
Female	51.4%	50.2%
Missing	4.9%	0.0%
Marital status		
Single/widowed/divorced	34.8%	41.1%
Married	59.9%	58.6%
Missing	5.3%	0.3%
Education		
No qualification/elementary/lower secondary school certificate	5.1%	26.2%
Secondary school certificate	21.7%	32.3%
University entrance qualification	25.8%	17.8%
University degree	41.6%	23.6%
Missing	5.8%	0.0%
Employment		
Employed	67.6%	66.3%
Not employed	27.3%	33.5%
Missing	5.1%	0.2%
Native language		
German	90.6%	96.5%
Other language	9.4%	3.5%
Missing		
Place of residence		
Up to 20 000 inhabitants	31.9%	36.7%
Up to 100 000 inhabitants	32.5%	33.1%
Over 100 000 inhabitants	35.6%	30.2%
Missing	0.0%	0.0%
Federal state		
Baden-Wuerttemberg	26.3%	25.3%
Berlin	15.5%	12.0%
North Rhine-Westphalia	22.3%	25.1%
Saxony-Anhalt	15.9%	15.5%
Schleswig-Holstein	19.9%	22.1%
Missing	0.0%	0.0%
Attitude "Skepticism towards digital technologies in healthcare"		
Mean value (standard deviation)^b^	3.1 (0.69)	3.1 (0.69)
Missing	7.0%	4.5%

Sample size *N* = 1 821; Complete cases *n* = 1 583

^a^Range of ages 18 to 80 years

^b^Range of scale from 1 "very low" to 5 "very high"

^c^Weighting by sex, age and educational attainment for a representative description, see Appendix Tables A2a, A2b and A2c. Cases missing data in any of these variables were assigned weights of 0

## Results

### Overall use of digitalized healthcare services

Table 2 describes the actual and prospective use of digitalized healthcare services and shows that although digitalized healthcare services were known, they were rarely actively used. Many participants stated that they would use such services under certain circumstances, which indicates that the potential of digitalized healthcare services has been recognized.

**Table 2:** Actual and prospective use of digitalized healthcare services

	**Unweighted**			**Weighted**^**a**^		
	**Relative frequency**			**Relative frequency**		
**Use of digitalized health services**	**Have used**	**Would use**	**Neither**	**Have used**	**Would use**	**Neither**
1. visit to the doctor for a concern outside of office hours (e.g., at night or at the weekend)	8.0%	66.5%	25.6%	8.7%	64.5%	26.8%
2. attend a training course on a specific disease (e.g., diabetes)	3.5%	77.0%	19.5%	4.6%	74.8%	20.7%
3. discuss a specific health problem with doctor or other medical personnel	6.4%	60.4%	33.3%	6.3%	62.4%	31.3%
4. follow-up examination after hospitalization	4.8%	55.9%	39.3%	5.5%	59.8%	34.7%
5. attend a medical appointment with a family member (children or parents in need of care)	5.6%	69.9%	24.5%	5.9%	71.4%	22.6%
6. examination of an illness that is not urgent (e.g., rash, cough, cold)	3.7%	60.2%	36.1%	4.2%	61.4%	34.4%
7. participation in psychological group therapy/psychotherapy	4.4%	26.4%	69.2%	4.0%	28.3%	67.7%
8. examination by a specialist in the event of an urgent problem (e.g., possible heart disease)	3.9%	40.2%	55.9%	3.8%	44.4%	51.8%
9. have prescriptions (e.g., for medication) sent to you	11.3%	83.6%	5.0%	10.6%	83.7%	5.7%
10. have sick note sent after examination	3.5%	83.1%	13.5%	3.0%	80.7%	16.2%
11. have sick note sent before examination	1.9%	56.2%	41.9%	1.9%	54.4%	43.7%

^a^Weighting by sex, age and educational attainment for a representative description, see Appendix Tables A2a, A2b and A2c

### Identification of user types

When looking at the different solutions with 2 to 6 classes, the models with 3 and 4 classes were selected as the best models according to the quality criteria (Table A3). However, the variant with only 3 classes offered the best interpretability and was therefore used for the description and the following multinomial regression analysis. The decision for the 3-class model was further supported by the matrix of the average latent class posterior probabilities (Table A4), showing that individuals were correctly classified into their respective latent classes with high accuracy.

The three classes were named "Potential", "Active" and "Rejecting". The latent class analysis was carried out with unweighted data; the frequencies of the class characteristics are listed below for the weighted sample (Table A5).

#### Type 1: Potential (53.8%)

Potential users are characterized by the fact that they would use most digitalized healthcare services in principle, but did not (yet) actively do so. Exceptions are participating in psychological group therapy/psychotherapy and examination by a specialist in the event of urgent problems, which are predominantly rejected. A small proportion of this group made very little use of a few services. These include visits to the doctor outside of consultation hours and digitally distributing prescriptions. All other services were almost not used.

#### Type 2: Active (18.3%)

Active respondents are characterized by the fact that they used digitalized healthcare services most frequently. The actual use of individual services by members of this group was between 10% and 35%. Here too, however, the rejection of a few services is not uncommon.

#### Type 3: Rejecting (27.9%)

Rejecting (non-)users are characterized by a predominantly negative attitude towards the majority of digitalized healthcare services, i.e., they neither used them nor would they want to use them.

**Fig. 1:**
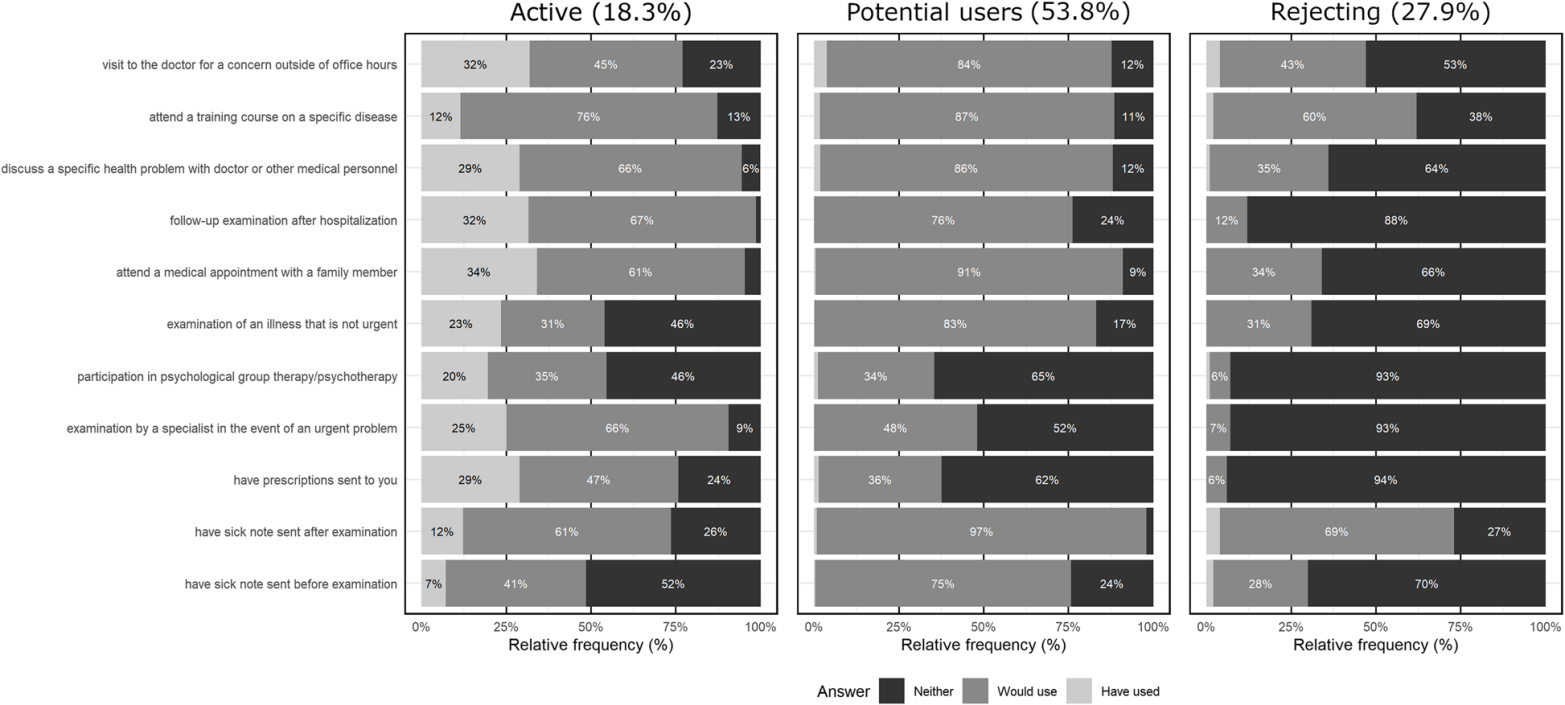
Class affiliation and response behavior regarding the use of digitalized healthcare services

Figure 1 shows the attitude towards and use of digitalized healthcare services depending on class.

### Characterization of user types

Multinomial logistic regressions were used to investigate the influence of different demographic variables and selected attitudinal variables on group membership. Table 3 shows the results with the group of rejecters as a reference. As an aid to interpretation, the Appendix contains the complementary models with the potential user group (Table A6a) and the active user group (Table A6b) as the respective reference group.

**Table 3:** Variables associated with the user types (multinomial logistic regressions, reference are the rejecting (non-)users

**Model 1**	**Potential**			**Active**		
	**OR**	**95% CI**	***p*-value**	**OR**	**95% CI**	***p*-value**
Age (standardized)^a^	0.67	0.57–0.78	<0.001***	0.82	0.67–1.00	0.050
Sex (Ref.: Male)						
Female	0.70	0.55–0.89	0.004**	0.73	0.52–1.01	0.054
Marital status (Ref: Single/widowed/divorced)						
Married	1.00	0.77–1.31	0.975	0.85	0.59–1.22	0.383
Education (Ref: Less than university degree)						
University degree	1.41	1.11–1.80	0.005**	0.67	0.47–0.94	0.020*
Native language (Ref.: German)						
Not German	0.85	0.49–1.52	0.599	1.16	0.75–3.03	0.245
Employment (Ref.: Not employed)						
Employed	1.05	0.78–1.42	0.752	0.50	0.34–0.73	<0.001***
Place of residence (Ref.: up to 20 000 inhabitants)						
Up to 100 000 inhabitants	0.94	0.71–1.25	0.664	1.15	0.78–1.69	0.482
Over 100 000 inhabitants	1.39	1.04–1.86	0.024*	1.31	0.87–1.95	0.193
**Model 2**	**Potential**			**Active**		
	**OR**	**95% CI**	***p*-value**	**OR**	**95% CI**	***p*-value**
Age (standardized)^a^	0.71	0.60–0.84	<0.001***	0.88	0.71–1.08	0.217
Sex (Ref.: Male)						
Female	0.76	0.59–0.99	0.040*	0.78	0.56–1.10	0.153
Marital status (Ref: Single/widowed/divorced)						
Married	0.93	0.70–1.24	0.632	0.77	0.53–1.13	0.183
Education (Ref: Less than university degree)						
University degree	1.41	1.08–1.82	0.011*	0.67	0.47–0.95	0.025*
Native language (Ref.: German)						
Not German	0.79	0.58–1.14	0.446	1.39	0.67–2.86	0.384
Employment (Ref.: Not employed)						
Employed	1.23	0.88–1.71	0.219	0.57	0.38–0.84	0.005**
Place of residence (Ref.: up to 20 000 inhabitants)						
Up to 100 000 inhabitants	0.88	0.64–1.20	0.419	0.96	0.63–1.44	0.833
Over 100 000 inhabitants	1.46	0.99–2.15	0.055	1.04	0.61–1.74	0.896
Skepticism^a^	0.39	0.34–0.45	<0.001***	0.46	0.38–0.55	<0.001***
Federal state (Ref.: Baden-Wuerttemberg)						
Berlin	0.72	0.44–1.19	0.203	1.02	0.52–1.96	0.964
North Rhine-Westphalia	0.82	0.56–1.19	0.293	1.12	0.70–1.80	0.639
Saxony-Anhalt	0.91	0.61–1.35	0.639	0.67	0.39–1.17	0.160
Schleswig-Holstein	0.99	0.68–1.43	0.940	0.72	0.43–1.18	0.194

^a^The odds ratio refers to one standard deviation (15.1 years for age and 0.69 for skepticism). *n* = 1 481; OR = odds ratio; 95% CI = 95% confidence interval; * = *p* < 0.05; ** = *p* < 0.01; *** = *p* < 0.001

The potential users were the youngest (46.8 years), had the highest level of education (28.1% with a university degree), were more likely to be male (51.5%) and had the highest proportion of employed respondents (75.0%). They were also least skeptical about digital technologies in healthcare. The active respondents had the lowest level of education (35.6%) and were the least likely to be employed (42.9%). With regard to digital technologies, they had a similarly positive attitude to the potential users. The proportion of women was highest among the rejecting (56.0%), while the other two groups were similar.

Sensitivity analyses did not reveal any significant differences when comparing the odds ratios of the multinomial logistic regression with those from separate logistic regressions. An alternative operationalization of education (university entrance qualification and higher = 1, lower/no degree = 0) showed equally directed correlations for those rejecting (OR = 1.23) and those actively participating (OR = 0.68). In addition, there were hardly any differences in the probability of belonging to the active (OR = 1.04) or the rejecting (OR = 1.10) group with increasing number of previous illnesses.

## Discussion

This study used latent class analysis to identify three user groups of digitalized healthcare services: potential (53.8%), active (18.3%) and rejecting (27.9%). Rejecting users stated that they had neither used nor wanted to use these services. The only exceptions to this were digitally sending prescriptions or sick notes. Potential users formed the largest group, who did not use many of the services but would be willing to do so. This group was the youngest, best educated and most frequently employed. The other two groups were more similar in terms of the characteristics examined.

It is surprising that the probability of belonging to the group of rejecters rather than the active was associated with a higher level of education. For both the TDI and general health-related applications higher school qualifications were associated with a higher probability of being an active user [10–20]. This finding can possibly be attributed to the healthcare services surveyed, which included a high proportion of general practitioner (GP) services, which in turn are more often used by people with a lower socioeconomic status [34]. In addition, it can be assumed that participants with a higher level of education are more likely to be healthy and therefore had no reason to make use of the available healthcare services at the time of the survey, but would be open to this if the need arose. However, the inclusion of possible pre-existing conditions did not confirm the assumption regarding the correlation between possible need due to health status and the use of digitalized healthcare.

Similar results were found for those in employment, who have a significantly higher chance of being among those who are rejecting rather than those who are actively using digitalized healthcare services. Additional research could provide new results if services such as examinations by a specialist or follow-up examinations after a hospital stay are firmly implemented and possibly facilitate utilization alongside employment.

As expected, rejecters had a higher probability of being skeptical towards digital technologies in the medical field. It should be noted that this includes attitudes towards treatment and improved therapies as well as data protection issues. A more detailed analysis could be carried out in order to identify specific concerns and thus starting points for improved use. Sensitivity analyses, in which the subdomains trust and concern have been added separately into the multinomial regression, showed the expected correlation and high significances. Because the general association between attitudes and the probability of belonging to one of the user groups did not change, we decided against using the subdomains. Further research could take a more detailed look at differences between the subdomains and their importance for the usage of digitalized healthcare services as well as the reason behind their mistrust and how this can be tackled.

The age differences correspond with the assumption that the use of digitalized healthcare services is based on need, explaining that younger people tend to belong to the group of potential users. Compared to those who reject digitalized healthcare services, those who are active are younger and less likely to be employed, which may be an indication that they have given up work, e.g., due to health problems. In addition, the combination of a lower level of education among active respondents may come into play here, according to which the primarily GP services surveyed here are more likely to be used by this group [34]. Older people were identified as both active and rejecting, but the rejecting group was significantly larger, comparable to the decrease in the likelihood of using general health-related applications with increasing age from previous findings [11–13, 16–18, 20, 21]. A possible future interest in digitalized healthcare services cannot be deduced from the present results, rather there were marginal differences, according to which the chance of belonging to the potential user group with increasing age was even lower than the probability of already active use.

It is surprising that the active users had the lowest level of education and at the same time a similarly positive attitude as the potential users. The positive attitude could be the decisive factor for active use. In addition, higher educational qualifications may be a protective factor that reduces the need to use healthcare services and most strongly predicts membership to the potential user group. It should also be mentioned that those in employment had a slightly higher probability of being among those who were potential users rather than those who were refusing, which may again be due to the selection options, which included electronic sick notes.

Gender-related differences can also be found in this study, although these are only significant for the potential user group. In general, women were less likely to be open-minded towards digitalized healthcare services or to actively use them. In conjunction with the inconsistent state of research, it is clear that further research into this aspect is required.

With regard to an urban-rural difference, there are similar, but only occasionally significant, results as for general health-related applications [13]: participants in large cities have a higher chance of belonging to the potential and active users than to the rejecting group. This may be due to the fact that working people and the younger ones with a higher level of education tend to live in larger cities [35]. This correlation disappears when attitudes towards digitalization in the healthcare sector are taken into account.

In contrast to our findings, the TDI identified five profiles [27, 29]. At the time of the survey, many of the healthcare services available for selection in this study were only being considered for implementation in the future and were only being used to a limited extent. Our results therefore presumably relate more to the process of dissemination before further types were developed. According to the TDI, it is most likely that the identified groups are *innovators* (active) and *early adopters* (potential). The extent to which the rejecters are more likely to develop into *laggards* or *non-users in the* course of the further spread of digitalized healthcare services requires further investigation. It might also be true, that both the TDI and our findings are representing a range of user types, which do not necessarily correspond. Further research could clarify whether changes in user types will occur with an increasing diffusion of digitalized healthcare services that correspond to the assumptions of the TDI or whether the user types found here will endure.

Further analyses should also look at other healthcare services, as many of the studies to date focus less on healthcare and more on preventive services [10]. This could be done taking the third level of the digital divide into account, which states that there are disparities in the ability to achieve an improved (health) outcome with the help of digital technologies [10]. Furthermore, investigating whether the differences in usage are more due to a third- or second-level digital divide could contribute to a better understanding of social differences and better care.

The practitioners' perspective could also provide additional insights into the extent to which certain patient groups would benefit from digitalized healthcare services and what obstacles providers see to this. This can be done, among other things, by incorporating Andersen's behavioral model, in which, in addition to socio-demographic factors, subjective or objective needs as well as enabling and inhibiting factors play a role in health behavior [36, 37]. A possible digital divide among practitioners should also be addressed, as they must also have both the technology and the skills to operate these technologies in order to be able to advise patients, prescribe suitable DiGAs and make adequate offers.

### Strengths and limitations

It is important to note that latent class analysis is an exploratory method and does not establish causality between the variables. However, it helps to identify different user groups and describe their characteristics. It cannot be ruled out that variables that could prove useful for characterizing the classes were not taken into account here. In addition, there could possibly be a responder bias due to the nature of the survey, as the results presented here are based on an online survey and certain people or groups who are less internet- or technology-savvy could therefore not be reached. During the planning phase of the study, attempts were made to counteract this by sending out information about the study by post. In addition, the sample was drawn on a population basis in five federal states. Older and socio-economically disadvantaged people in particular were underrepresented, which is why a weighting was applied in the description. Additionally, the presented findings are based on cross-sectional data. Therefore, possible changes due to innovations or a more substantial understanding of the population for the advantages of digitalized healthcare as well es a causal relationship can’t be depicted. The results of this study must also be reviewed by means of a new survey to ensure that they are up to date, as digitalization has also progressed and been discussed in the medical field since the data was collected. In addition, the survey was conducted during the first wave of the coronavirus pandemic. During the survey period, there was a high level of uncertainty regarding the usage of medical services, because of fear of infections. An increasing willingness to use digitalized healthcare services was met with a lack of preparation of the infrastructure and a general overload of the healthcare system. Therefore, it is necessary to check whether the open-mindedness towards digitalized healthcare services also exists after the return to regular care.

## Conclusions

In this study, three user types were identified that differ in terms of their characteristics. Active users tend to be older, not employed, less likely to be highly educated and less skeptical of digital innovations in healthcare. Rejecting users are very similar to active users, only more likely to be female, slightly higher educated and characterized by a clear skepticism towards digital innovations in healthcare. Potential users are the most clearly defined and are on average younger, more educated and more likely to be employed than the other two types. The results presented here can be used when offering planning digitalized healthcare services. By providing information on user types, access can be made more targeted and, if necessary, simplified in order to reach all relevant groups of people. In addition, the information on the relationship between attitudes and likelihood of use can provide a basis for information material for the expansion of digitalized healthcare services.

It remains to be seen whether this will expand to all five types of TDI with a higher prevalence of digitalized healthcare services. In addition, further medical services should be surveyed and additional features of the characterization should be included in order to be able to determine whether the different utilization is due to the user structure or rather on the provider side.

## Supplementary Information

Supplementary Material 1.

## Data Availability

The datasets used and/or analyzed during the current study are available from the corresponding author on reasonable request.

## Abbreviations

DiGA: Digital health application, German: Digitale Gesundheitsangebote
ePa: The electronic patient record, German: elektronische Patientenakte
GP: General practitioner
HeReCa: Health Related Beliefs and Health Care Experiences in Germany
LCA: Latent class analysis
OR: Odds Ratio
TDI: Theory of the diffusion of innovations
