# Supplementary material for: User profiles in digitalized healthcare: active, potential, and rejecting — a cross-sectional study using latent class analysis
Source: BMC Health Serv Res. 2024 Sep 17;24:1083. doi: 10.1186/s12913-024-11523-w (PMC11409736; doi:10.1186/s12913-024-11523-w)
Supplement: Supplementary file 1 — Supplementary Material 1. [file 12913_2024_11523_MOESM1_ESM.docx]

Supplementary material

**Table A1. Attitudinal questions regarding the opportunities and risks of digital technologies in healthcare**

| **Item** | **Question "How do you rate..."** |
| --- | --- |
| 1 | ... your trust in the medical opportunities offered by new digital technologies? (e.g., care in rural areas) |
| 2 | ... your trust in the organizational and administrative opportunities offered by the use of digital technologies? (e.g., scheduling, cost savings) |
| 3 | ... your trust in the organizational and administrative opportunities offered by the use of digital technologies? (e.g., scheduling, cost savings) |
| 4 | ... your trust that large amounts of digital data ("big data") will improve the healthcare system? |
| 5 | ... your concern about possible misuse of data protection when using digital technologies in the healthcare sector? |
| 6 | ... your concern about becoming a "transparent" patient? |
| 7 | ... your concern regarding an overall negative development in the healthcare sector due to the use of digital technologies? (e.g., wrong decisions due to incorrect data collection and transfer, fewer human contacts due to orientation towards artificial intelligence) |
| 8 | ... your concern that the health apps are not sufficiently tested and therefore good quality cannot be guaranteed. |

The response options consisted of a five-point Likert scale from 1 "very low" to 5 "very high".

**Table A2a: Relative frequencies (%) representative Destatis sample (n=64 842)^a^**

|  |  | **Age** |  |  |  |  |  |  |
| --- | --- | --- | --- | --- | --- | --- | --- | --- |
| **Sex** | **School-leaving certificate^b^** | **15-24** | **25-34** | **35-44** | **45-54** | **55-64** | **65+** | **Total** |
| Female | Lower secondary (elementary) school | 0.4 | 0.8 | 1.1 | 1.5 | 2.4 | 7.4 | 13.6 |
|  | Secondary school certificate or equivalent (incl. POS) | 1.3 | 2.1 | 2.5 | 3.5 | 4.1 | 4.5 | 18 |
|  | Advanced technical college or university entrance qualification | 2.4 | 4.6 | 3.9 | 3.2 | 2.9 | 2.4 | 19.3 |
|  | Total | 4.1 | 7.5 | 7.5 | 8.2 | 9.4 | 14.2 | 50.9 |
| Male | Lower secondary (elementary) school | 0.7 | 1.3 | 1.6 | 2.1 | 2.8 | 5.3 | 13.9 |
|  | Secondary school certificate or equivalent (incl. POS) | 1.6 | 2.2 | 2.3 | 2.9 | 3.3 | 3 | 15.3 |
|  | Advanced technical college or university entrance qualification | 2.1 | 4.3 | 3.6 | 3.3 | 3.2 | 3.3 | 19.9 |
|  | Total | 4.4 | 7.9 | 7.6 | 8.3 | 9.3 | 11.7 | 49.1 |

**Table A2b: Relative frequencies (%) HeReCa sample (n=1 732)^c^**

|  |  | **Age** |  |  |  |  |  |  |
| --- | --- | --- | --- | --- | --- | --- | --- | --- |
| **Sex** | **School-leaving certificate^b^** | **15-24** | **25-34** | **35-44** | **45-54** | **55-64** | **65+** | **Total** |
| Female | Lower secondary (elementary) school | 0 | 0.2 | 0 | 0.3 | 0.8 | 0.8 | 2.1 |
|  | Secondary school certificate or equivalent (incl. POS) | 0.2 | 0.5 | 1.2 | 3.9 | 5.4 | 3.5 | 14.8 |
|  | Advanced technical college or university entrance qualification | 2.4 | 6.9 | 6.9 | 7.5 | 8.2 | 4.2 | 36.1 |
|  | Total | 2.6 | 7.6 | 8.1 | 11.7 | 14.4 | 8.5 | 53 |
| Male | Lower secondary (elementary) school | 0.2 | 0.3 | 0.3 | 0.2 | 0.9 | 1.3 | 3.3 |
|  | Secondary school certificate or equivalent (incl. POS) | 0.2 | 0.6 | 0.6 | 2.7 | 2.7 | 2.3 | 9.2 |
|  | Advanced technical college or university entrance qualification | 1.4 | 4.2 | 4 | 6.5 | 7.3 | 9.5 | 32.8 |
|  | Total | 1.8 | 5 | 5 | 9.4 | 10.9 | 13.1 | 45.3 |

**Table A2c: Weighting for HeReCa sample**

|  |  | **Age** |  |  |  |  |  |  |
| --- | --- | --- | --- | --- | --- | --- | --- | --- |
| **Sex** | **School-leaving certificate^b^** | **15-24** | **25-34** | **35-44** | **45-54** | **55-64** | **65+** | **Total** |
| Female | Lower secondary (elementary) school | - | 3 432 | - | 5 271 | 2 966 | 8 979 | 6 276 |
|  | Secondary school certificate or equivalent (incl. POS) | 5 361 | 3 923 | 2 055 | 875 | 748 | 1 269 | 1 195 |
|  | Advanced technical college or university entrance qualification | 997 | 654 | 553 | 416 | 342 | 554 | 525 |
|  | Total | 1 540 | 961 | 904 | 689 | 642 | 1 648 | 944 |
| Male | Lower secondary (elementary) school | 3 163 | 4 509 | 4 633 | 8 728 | 3 213 | 3 909 | 4 140 |
|  | Secondary school certificate or equivalent (incl. POS) | 6 746 | 3 735 | 3 591 | 1 061 | 1 182 | 1 297 | 1 642 |
|  | Advanced technical college or university entrance qualification | 1 478 | 1 026 | 885 | 502 | 430 | 348 | 596 |
|  | Total | 2 347 | 1 537 | 1 486 | 865 | 840 | 876 | 1 066 |

Source: <https://www.datenportal.bmbf.de/portal/de/Tabelle-0.17.html>

Notes: (a) After excluding the category "Not specified or no school-leaving qualification" (n=). (b) Separate categories for polytechnic secondary schools in the GDR and secondary school or equivalent qualification collapsed into one category. (c) After excluding cases in the categories "student, attending a general school (full-time or part-time)" (n=6), "another school-leaving qualification" (n=18) and missing values (n=88).

**Table A3: Quality criteria of the latent class analyses with different numbers of classes**

| Number of classes | LL | AIC | BIC | Χ^2^ | Entropy R^2^ |
| --- | --- | --- | --- | --- | --- |
| 2 | -11394.21 | 22892.41 | 23170 | 48726652.8 | 0.80 |
| 3 | -10788.69 | 21741.38 | 22179.12 | 3711232.08 | 0.81 |
| 4 | -10540.79 | 21305.58 | 21903.46 | 2401789.1 | 0.80 |
| 5 | -10466.59 | 21217.17 | 21975.2 | 2238412.76 | 0.76 |
| 6 | -12372.82 | 25089.63 | 26007.81 | 141927265 | 1 |

Notes: LL = Log Likelihood; AIC = Akaike Information Criterion; BIC = Bayesian Information Criterion; Χ2 = Chi-square goodness of fit. Bold = best fit for the respective statistic with a simultaneous R2 value of > 0.8 (lower values indicate a better fit for each of the likelihood-based statistics).

**Table A4. Matrix of the average latent class posterior probability**

|  | **Active** | **Rejecting** | **Potential** |
| --- | --- | --- | --- |
| **Active** | 92.1 % | 0.4 % | 7.5 % |
| **Rejecting** | 1.3 % | 92.5 % | 6.2 % |
| **Potential** | 3.9 % | 3.0 % | 93.1 % |

**Table A5: Class description (weighted)**

| **Feature** | **Active** | **Potential** | **Rejecting** |
| --- | --- | --- | --- |
| Age |  |  |  |
| Mean value (standard deviation)^a^ | 52.0 (17.1) | 46.8 (16.0) | 53.7 (15.9) |
| Sex |  |  |  |
| Male | 53.2 % | 51.4 % | 44.0 % |
| Female | 46.8 % | 48.6 % | 56.0 % |
| Marital status |  |  |  |
| Single/widowed/divorced | 40.1 % | 46.8 % | 35.6 % |
| Married | 59.9 % | 53.2 % | 64.4 % |
| Education |  |  |  |
| No qualification/elementary/lower secondary school certificate | 35.6 % | 20.6 % | 28.7 % |
| Secondary school certificate/POS | 31.5 % | 32.4 % | 33.6 % |
| University entrance qualification | 17.8 % | 18.8 % | 16.3 % |
| University degree | 15.1 % | 28.1 % | 21.4 % |
| Employment |  |  |  |
| Employed | 57.1 % | 75.0 % | 60.9 % |
| Not employed | 42.9 % | 25.0 % | 39.1 % |
| Native language |  |  |  |
| German | 92.5 % | 97.2 % | 97.3 % |
| Other language | 7.5 % | 2.8 % | 2.7 % |
| Place of residence |  |  |  |
| Up to 20 000 inhabitants | 36.4 % | 36.5 % | 46.4 % |
| Up to 100 000 inhabitants | 33.8 % | 29.0 % | 28.5 % |
| Over 100 000 inhabitants | 29.8 % | 34.5 % | 25.1 % |
| Federal state |  |  |  |
| Baden-Württemberg | 29.2 % | 26.2 % | 23.1 % |
| Berlin | 10.9 % | 13.3 % | 10.7 % |
| North Rhine-Westphalia | 31.2 % | 21.1 % | 22.7 % |
| Saxony-Anhalt | 10.5 % | 17.1 % | 16.5 % |
| Schleswig-Holstein | 18.2 % | 22.4 % | 26.9 % |
| Attitude "Skepticism towards digital technologies in healthcare" |  |  |  |
| Mean value (standard deviation)^b^ | 3.0 (0.68) | 3.0 (0.64) | 3.5 (0.61) |

Notes: Sample size N=1 821; complete cases n=1 481; weighting by sex, age and educational attainment for a representative description, see Appendix Tables A1a, A1b and A1c. ^a^ Age range 15 to 80 years; ^b^ Scale range from 1 "very low" to 5 "very high".

**Table A6a. Variables associated with the user types (multinomial logistic regressions, reference are the potential)**

| **Model 1** | **Rejecting** | | | | **Active** | | | |
| --- | --- | --- | --- | --- | --- | --- | --- | --- |
| **Indicator** | **OR** | **95% CI** | **p-value** |  | **OR** | **95% CI** | **p-value** |  |
| Age (z-standardized) | 1.50 | 1.29 - 1.74 | 0.000 | *** | 1.23 | 1.03 - 1.46 | 0.026 | * |
| Sex  (Ref.: Male) |  |  |  |  |  |  |  |  |
| Female | 1.43 | 1.12 - 1.82 | 0.004 | ** | 1.04 | 0.77 - 1.40 | 0.811 |  |
| Marital status  (Ref: Single/widowed/divorced) |  |  |  |  |  |  |  |  |
| Married | 1.00 | 0.76 - 1.30 | 0.975 |  | 0.85 | 0.60 - 1.19 | 0.335 |  |
| Education  (Ref: All up to university entrance qualification) |  |  |  |  |  |  |  |  |
| University degree | 0.71 | 0.56 - 0.90 | 0.005 | ** | 0.47 | 0.34 - 0.65 | 0.000 | *** |
| Native language  (Ref.: German) |  |  |  |  |  |  |  |  |
| Not German | 1.16 | 0.66 - 2.04 | 0.599 |  | 1.76 | 0.93 - 3.33 | 0.081 |  |
| Employment  (Ref.: Not employed) |  |  |  |  |  |  |  |  |
| Employed | 0.95 | 0.70 - 1.29 | 0.752 |  | 0.48 | 0.34 - 0.68 | 0.000 | *** |
| Place of residence  (Ref.: size up to 20 000 inhabitants) |  |  |  |  |  |  |  |  |
| City over 20 000 inhabitants | 1.07 | 0.80 - 1.42 | 0.664 |  | 1.22 | 0.85 - 1.76 | 0.279 |  |
| City with over 100 000 inhabitants | 0.72 | 0.54 - 0.96 | 0.024 | * | 0.94 | 0.65 - 1.36 | 0.729 |  |
|  | | | | | | | | |
| **Model 2** | **Rejecting** | | | | **Active** | | | |
| **Indicator** | **OR** | **95% CI** | **p-value** |  | **OR** | **95% CI** | **p-value** |  |
| Age (z-standardized) | 1.40 | 1.19 - 1.65 | 0.000 | *** | 1.23 | 1.02 - 1.47 | 0.026 | * |
| Sex  (Ref.: Male) |  |  |  |  |  |  |  |  |
| Female | 1.31 | 1.01 - 1.70 | 0.040 | * | 1.03 | 0.76 - 1.39 | 0.868 |  |
| Marital status  (Ref: Single/widowed/divorced) |  |  |  |  |  |  |  |  |
| Married | 1.07 | 0.80 - 1.43 | 0.632 |  | 0.83 | 0.59 - 1.17 | 0.287 |  |
| Education  (Ref: All up to university entrance qualification) |  |  |  |  |  |  |  |  |
| University degree | 0.71 | 0.55 - 0.92 | 0.011 | * | 0.47 | 0.34 - 0.65 | 0.000 | *** |
| Native language  (Ref.: German) |  |  |  |  |  |  |  |  |
| Not German | 1.26 | 0.68 - 2.33 | 0.446 |  | 1.75 | 0.92 - 3.33 | 0.089 |  |
| Employment  (Ref.: Not employed) |  |  |  |  |  |  |  |  |
| Employed | 0.81 | 0.59 - 1.13 | 0.219 |  | 0.46 | 0.32 - 0.66 | 0.000 | *** |
| Place of residence  (Ref.: size up to 20 000 inhabitants) |  |  |  |  |  |  |  |  |
| City over 20 000 inhabitants | 1.14 | 0.83 - 1.56 | 0.419 |  | 1.09 | 0.74 - 1.59 | 0.660 |  |
| City with over 100 000 inhabitants | 0.68 | 0.46 - 1.01 | 0.055 |  | 0.71 | 0.45 - 1.12 | 0.141 |  |
| Skepticism | 2.54 | 2.20 - 2.92 | 0.000 | *** | 1.16 | 0.99 - 1.37 | 0.064 |  |
| Federal state  (Ref.: Baden-Würtemberg) |  |  |  |  |  |  |  |  |
| Berlin | 1.38 | 0.84 - 2.28 | 0.203 |  | 1.41 | 0.79 - 2.50 | 0.249 |  |
| North Rhine-Westphalia | 1.23 | 0.84 - 1.80 | 0.293 |  | 1.37 | 0.90 - 2.10 | 0.143 |  |
| Saxony-Anhalt | 1.10 | 0.74 - 1.64 | 0.639 |  | 0.74 | 0.45 - 1.23 | 0.248 |  |
| Schleswig-Holstein | 1.01 | 0.70 - 1.47 | 0.940 |  | 0.73 | 0.46 - 1.15 | 0.172 |  |

Notes: n = 1 481; OR = odds ratio; 95% CI = 95% confidence interval; * = p<0.05; ** = p<0.01; *** = p<0.001.

**Table A6b. Variables associated with the user types (multinomial logistic regressions, reference are the active users)**

| **Model 1** | **Potential** | | | | **Rejecting** | | | |
| --- | --- | --- | --- | --- | --- | --- | --- | --- |
| **Indicator** | **OR** | **95% CI** | **p-value** |  | **OR** | **95% CI** | **p-value** |  |
| Age (standardized) | 0.82 | 0.68 - 0.98 | 0.026 | * | 1.22 | 1.00 - 1.49 | 0.050 |  |
| Sex  (Ref.: Male) |  |  |  |  |  |  |  |  |
| Female | 0.96 | 0.71 - 1.30 | 0.811 |  | 1.38 | 0.99 - 1.91 | 0.054 |  |
| Marital status  (Ref: Single/widowed/divorced) |  |  |  |  |  |  |  |  |
| Married | 1.18 | 0.84 - 1.66 | 0.335 |  | 1.18 | 0.82 - 1.69 | 0.383 |  |
| Education  (Ref: All up to university entrance qualification) |  |  |  |  |  |  |  |  |
| University degree | 2.12 | 1.54 - 2.91 | 0.000 | *** | 1.50 | 1.07 - 2.11 | 0.020 | * |
| Native language  (Ref.: German) |  |  |  |  |  |  |  |  |
| Not German | 0.56 | 0.30 - 1.08 | 0.081 |  | 0.66 | 0.33 - 1.33 | 0.245 |  |
| Employment  (Ref.: Not employed) |  |  |  |  |  |  |  |  |
| Employed | 2.10 | 1.48 - 2.97 | 0.000 | *** | 2.00 | 1.36 - 2.92 | 0.000 | *** |
| Place of residence  (Ref.: size up to 20 000 inhabitants) |  |  |  |  |  |  |  |  |
| City over 20 000 inhabitants | 0.82 | 0.57 - 1.18 | 0.279 |  | 0.87 | 0.59 - 1.28 | 0.482 |  |
| City with over 100 000 inhabitants | 1.07 | 0.74 - 1.54 | 0.729 |  | 0.77 | 0.51 - 1.14 | 0.193 |  |
|  | | | | | | | | |
| **Model 2** | **Potential** | | | | **Rejecting** | | | |
| **Indicator** | **OR** | **95% CI** | **p-value** |  | **OR** | **95% CI** | **p-value** |  |
| Age (z-standardized) | 0.81 | 0.68 - 0.98 | 0.026 | * | 1.14 | 0.93 - 1.4 | 0.217 |  |
| Sex  (Ref.: Male) |  |  |  |  |  |  |  |  |
| Female | 0.97 | 0.72 - 1.32 | 0.868 |  | 1.28 | 0.91 - 1.79 | 0.153 |  |
| Marital status  (Ref: Single/widowed/divorced) |  |  |  |  |  |  |  |  |
| Married | 1.20 | 0.85 - 1.70 | 0.287 |  | 1.29 | 0.89 - 1.89 | 0.183 |  |
| Education  (Ref: All up to university entrance qualification) |  |  |  |  |  |  |  |  |
| University degree | 2.11 | 1.53 - 2.91 | 0.00 | *** | 1.50 | 1.05 - 2.14 | 0.025 | * |
| Native language  (Ref.: German) |  |  |  |  |  |  |  |  |
| Not German | 0.57 | 0.30 - 1.09 | 0.089 |  | 0.72 | 0.35 - 1.49 | 0.384 |  |
| Employment  (Ref.: Not employed) |  |  |  |  |  |  |  |  |
| Employed | 2.17 | 1.52 - 3.08 | 0.000 | *** | 1.76 | 1.18 - 2.62 | 0.005 | ** |
| Place of residence  (Ref.: size up to 20 000 inhabitants) |  |  |  |  |  |  |  |  |
| City over 20 000 inhabitants | 0.92 | 0.63 - 1.34 | 0.660 |  | 1.05 | 0.69 - 1.58 | 0.833 |  |
| City with over 100 000 inhabitants | 1.41 | 0.89 - 2.23 | 0.141 |  | 0.97 | 0.57 - 1.63 | 0.896 |  |
| Skepticism | 0.86 | 0.73 - 1.01 | 0.064 |  | 2.18 | 1.82 - 2.61 | 0.000 | *** |
| Federal state  (Ref.: Baden-Würtemberg) |  |  |  |  |  |  |  |  |
| Berlin | 0.71 | 0.40 - 1.27 | 0.249 |  | 0.99 | 0.51 - 1.91 | 0.964 |  |
| North Rhine-Westphalia | 0.73 | 0.48 - 1.11 | 0.143 |  | 0.89 | 0.55 - 1.44 | 0.639 |  |
| Saxony-Anhalt | 1.35 | 0.81 - 2.23 | 0.248 |  | 1.48 | 0.86 - 2.57 | 0.160 |  |
| Schleswig-Holstein | 1.38 | 0.87 - 2.17 | 0.172 |  | 1.40 | 0.84 - 2.31 | 0.194 |  |

Notes: n = 1 481; OR = odds ratio; 95% CI = 95% confidence interval; * = p<0.05; ** = p<0.01; *** = p<0.001.
